# CircASPH promotes KGN cells proliferation through miR‐375/MAP2K6 axis in Polycystic Ovary Syndrome

**DOI:** 10.1111/jcmm.16231

**Published:** 2020-12-28

**Authors:** Gengxiang Wu, Jing Xia, Zhe Yang, Yajie Chen, Wei Jiang, Tailang Yin, Jing Yang

**Affiliations:** ^1^ Reproductive Medical Centre Renmin Hospital of Wuhan University Wuhan China

**Keywords:** circASPH, KGN cells, MAP2K6, miR‐375, polycystic ovary syndrome

## Abstract

Polycystic Ovary Syndrome (PCOS) is a kind of endocrine disorder which is prevalent in adult women, so exploring more biomarkers for PCOS is imperative. Recently, circular RNA and microRNA are confirmed to be related with PCOS development. Whether circular RNA ASPH (circASPH) is involved in PCOS need to be studied further. We utilized RT‐qPCR to measure the expression levels of circASPH, miR‐375 and MAP2K6 in PCOS patients and normal group. The effects of circASPH and miR‐375 on KGN cells proliferation and apoptosis were observed by CCK‐8 assay, EdU incorporation assay and apoptosis assay, separately. Then Dual‐luciferase reporter assay was carried out to verify the circASPH/miR375 axis and miR375/MAP2K6 axis. The interaction between circASPH and MAP2K6 were detected with the support of RT‐qPCR and Western blot. We found circASPH and MAP2K6 were both over‐expressed in PCOS patients, while miR‐375 was in the opposite direction. Moreover, miR‐375 was negatively regulated by circASPH, while MAP2K6 was positively regulated by circASPH. In addition, circASPH directly targeted miR‐375, which targeted MAP2K6. More than that, the knockdown of circASPH repressed KGN cells proliferation and enhanced apoptosis, while the silence of miR‐375 reversed the above effects. In conclusion, circASPH promotes KGN cells proliferation through miR‐375/MAP2K6 axis in PCOS, and they are thought‐provoking biomarkers for PCOS diagnosis and therapy.

## 1 INTRODUCTION

Polycystic Ovary Syndrome (PCOS) is the most regular endocrine disorder in adult women with the occurrence of 6%‐12% in overall women.^1^ Clinical characteristics are multifarious, mainly manifested as hyperandrogenism, oligomenorrhea, ovarian polycystic changes, anovulation, infertility and so on. The women who was affected with PCOS usually have an additive risk suffering from type II diabetes mellitus, metabolic diseases, hypertension and cardiovascular events, and so on.^2^, ^3^, ^4^ In addition, the risk of suffering from PCOS could be increased by obesity, glucose intolerance, dyslipidaemia and arterial hypertension.^4^, ^5^ As a syndrome, the pathogenesis of PCOS is complicated and the studies are always underway for discovering diagnosis and treatment approach. Due to the development of high‐throughput sequencing technologies, regulatory genes participating in the pathogenesis of PCOS have been discovered successively in previous studies.^6^, ^7^

Circular RNA is a class of endogenous, single‐strand, circular non‐coding RNA without 5′‐3′ polarity, and it is the byproduct of wrong‐splicing of precursor mRNA (pre‐mRNA) in eukaryotes. Circular RNA have been certificated to regulate gene in transcriptional or post‐transcriptional level by sponging microRNA mainly and play vital roles in a series of physiological processes.^8^, ^9^, ^10^ Recently, the studies focused on the effects of circular RNA in PCOS development are growing vigorously, hundreds of circular RNA have been found dysregulated in PCOS model.^11^, ^12^, ^13^ Moreover, the detailed regulatory effects of circular RNA have also been excavated, such as circ_0023942 exerted as a negative role in ovarian granulosa cell proliferation.^14^ Earlier, circASPH was reported to act as a tumour‐promotor through targeting miR‐370/HMGA2 axis in lung adenocarcinoma,^15^ however, the potential role of circASPH in PCOS is still unclear.

As the commonest gene regulating factor, microRNAs have also been considered in the pathological of PCOS, and many microRNAs were investigated to be correlated with PCOS [5‐1,5‐5]. Further, miR‐19b, miR‐145, miR‐29a, miR‐204 were all validated to be a negative regulator in PCOS cell proliferation.^16^, ^17^, ^18^, ^19^ Meanwhile, several circRNA/miRNA axis were pointed out, including circ‐PUM1/miR‐760 and circ_0118530/miR‐136.^20^, ^21^ The knowledge of circASPH sponged microRNA in PCOS development is still lacking.

In our study, we speculated that circASPH is crucial in the processes of PCOS. First, the experiment results showed that circASPH was up‐regulated in PCOS. Then, bioinformatic analysis was applied to predict the downstream regulator of circASPH and miR‐375 was one of the miRNA response elements. Moreover, we found that the up‐regulation of circASPH enhanced KGN cells proliferation through targeting miR‐375/MAP2K6 axis. Our findings partially demonstrate the role of circASPH/miR‐375/MAP2K6 axis in PCOS, which may be helpful for PCOS clinical diagnose and therapy.

## 2 METHODS

### 2.1 Clinical samples

Five patients with PCOS were enrolled from Renmin Hospital of Wuhan University. Nine women without PCOS at the similar age as that of PCOS patients were gathered as the normal control group. The diagnosis of PCOS is established by the presence of at least two out of three of the following criteria: (1) signs of clinical or biochemical hyperandrogenism, (2) chronic ovulatory dysfunction and (3) polycystic ovarian morphology. All patients offered the written informed consent of the following study, and all the experiments followed by the local ethics committee of Renmin Hospital of Wuhan University. Ovarian granulosa cells were obtained from all 14 samples using follicular aspiration of mature oocytes. The clear follicular fluid containing granulosa cells was collected and centrifuged at 626 *g* for 10 minutes. The cells were resuspended in hyaluronidase and digested at 37°C for 20 minutes. Next, we added lymphocyte separation liquid and centrifuged at 626 *g* for 10 minutes. The intermediate white cloudy cell clusters were granulosa cells.

### 2.2 Cells culture and transfection

KGN and SVOG cells (GC cell line) were bought from Cell Bank of the Chinese Academy of Science (Shanghai, China). All cells were maintained in DMEM medium containing 10% FBS (Gibco, USA) and 1% gentamicin, and all cells were incubated under a 5% CO_2_, humidified atmosphere and 37°C condition. All plasmids, si RNAs and their negative controls purchased from GeneChem (Shanghai, China), were transfected using lipofectamine 3000 reagent (Invitrogen, USA) followed by the manufacturer's instructions.

### 2.3 RT‐qPCR

Total RNA were lysed from ovarian granulosa cells and GC cells with the help of Trizol abided by the protocol. Then RNAs were reverse‐transcript into cDNA using SYBR Premix Ex Taq II kit (TaKaRa, Japan). GAPDH was acted as an internal control. Target RNAs were amplified on 7500 Real‐Time PCR system (Applied Biosystem, USA) using SYBR Green PCR Master Mix. The values were determined by using the 2^−ΔΔCT^ method.

### 2.4 Dual‐luciferase reporter assay

The circASPH WT, MAP2K6 WT and their MUT plasmids were constructed by GeneChem (Shanghai, China). The plasmids contained circASPH WT and circASPH MUT along with miR‐375 mimic and their negative controls, MAP2K6 WT and MAP2K6 MUT along with miR‐375 mimic and their negative controls, MAP2K6 WT and MAP2K6 MUT along with OE circASPH and their negative controls, were co‐transfected into KGN cells separately. Finally, we used Dual‐luciferase reporter assay Kit (Promega) to observe the luciferase reporter activity.

### 2.5 CCK‐8 assay

Cell Counting Kit‐8 (CCK‐8) assay was employed to measure the proliferation ability of GC cells. We used CCK‐8 solution to count the cell numbers at 450 nm following the protocol. The data were gathered every 24 hours from 0 to 96 hours respectively.

### 2.6 EdU assay

A Cell‐Light EdU DNA Cell proliferation Kit (RiboBio, China) was used to test the cell proliferation. After KGN cells transfected with OE circASPH, OE NC, si circASPH and si NC for 2 days, EdU were added for 48 hours. Then the cells were fixed using 4% paraformaldehyde (PFA) and stained by Apollo Dye Solution. Nucleic acids were stained by DAPI. A fluorescence microscope and Image‐Pro Plus software were utilized to take images and assess cells EdU incorporation.

### 2.7 Apoptosis assay

Propidium iodide (PI) and Annexin V‐conjugated Fluorescein isothiocynate (Annexin V‐FITC) were used to assess the apoptotic cells rate. Transfected KGN cells were cultured with PI and Annexin V‐FITC for 20 minutes without light. Cell apoptosis was assessed with the help of Flow cytometry (FC). The data of apoptotic cells rate were measured by FlowJo software (Tree Star, USA).

### 2.8 Western blot

Transfected KGN cells with OE circASPH, OE NC, si circASPH, and si NC were lysed using RIPA buffer to obtain proteins, which were separated via 10% SDS‐PAGE gels and then moved to PVDF membrane (Thermo Fisher Scientific, USA). After blocking the above membranes in 5% dried skim milk at 25°C for 1 hour, the membranes were indicated with specific primary antibodies (MAP2K6, ab154901; Cleaved PARP, ab32064, Cleaved Caspase 3, ab32042; GAPDH, ab9485) at 4°C overnight. After washing with TBST three times and the membranes were incubated with secondary antibodies for 2 hours at 25°C. All antibodies used were purchased from Abcam (USA). We used a BCA Protein Quantitation Kit (Beyotime Institute of Biotechnology) to quantify proteins.

### 2.9 Statistical analysis

All data were exhibited as mean ± standard deviation (SD) and were the average of three experiments. We carried out Student's *t* test and one‐way ANOVA analysis to measure the differences between quantitative variables. All statistical analysis were performed with the help of R studio (3.6.1). A two‐tailed *P*‐value < 0.05 was considered to be statistically significant.

## 3 RESULTS

### 3.1 CircASPH was over‐expressed, while miR‐375 was down‐regulated in PCOS

**FIGURE 1:**
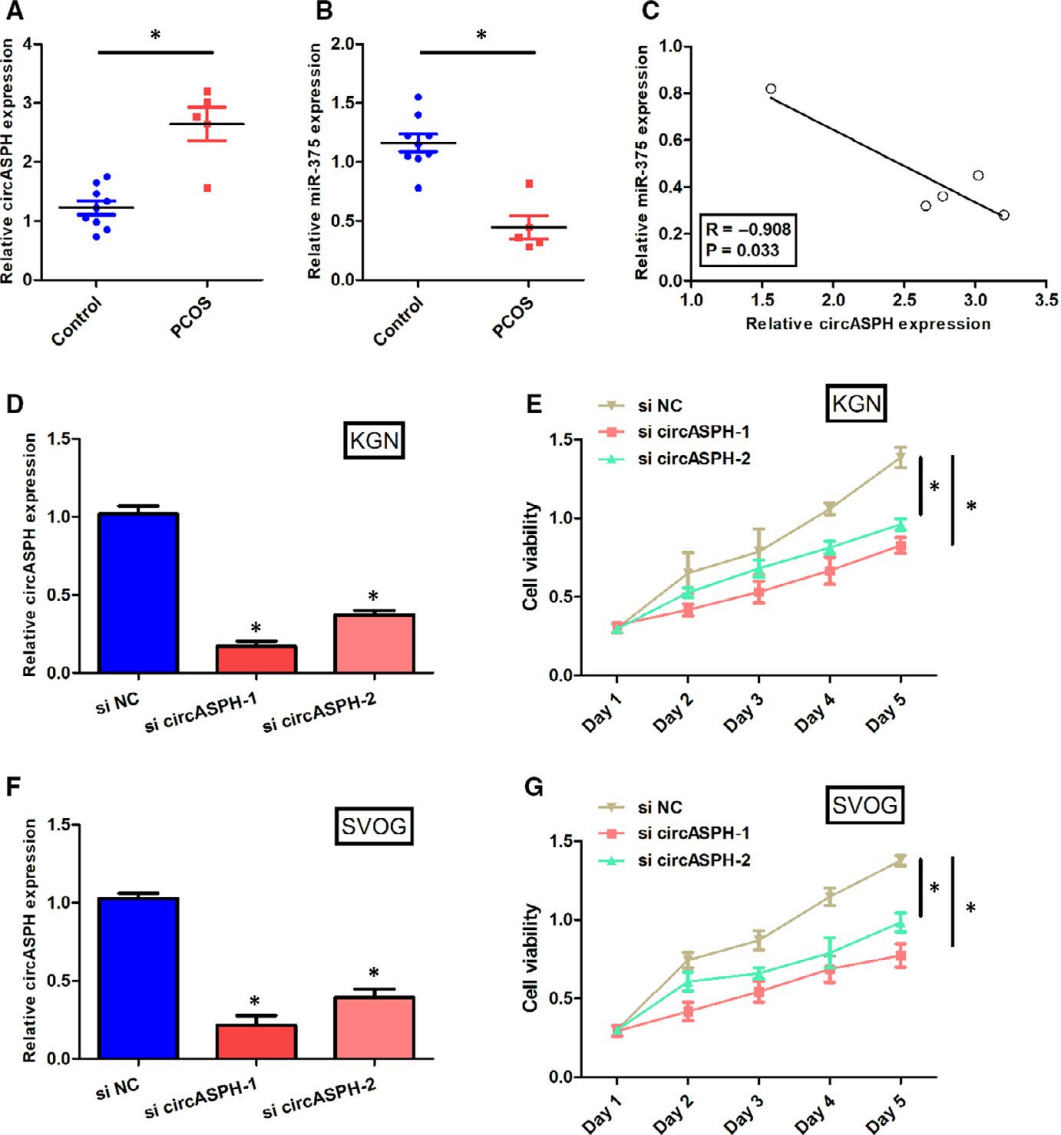
CircASPH was over‐expressed, while miR‐375 was down‐regulated in PCOS. A, The expression levels of circASPH in five PCOS patients and nine non‐PCOS group. B, The expression levels of miR‐375 in five PCOS patients and nine non‐PCOS Control group. C, The linear regression between circASPH expression and miR‐375 expression in five PCOS patients and nine non‐PCOS Control group. D, The expression levels of circASPH in KGN cells transfected with si circASPH‐1, si circASPH‐2 and si NC. E, The cell viability of KGN cells transfected with si circASPH‐1, si circASPH‐2 and si NC at Day 1, Day 2, Day 3, Day 4 and Day 5. F, The expression levels of circASPH in SVOG cells transfected with si circASPH‐1, si circASPH‐2 and si NC. G, The cell viability of SVOG cells transfected with si circASPH‐1, si circASPH‐2 and si NC at Day 1, Day 2, Day 3, Day 4 and Day 5. All quantitative values were the average of three independent experiments. *P < 0.05 [Colour figure can be viewed at wileyonlinelibrary.com]

By a Starbase v2.0 prediction, circASPH shared potential miRNA response elements for multiple miRNAs such as miR‐1182, miR‐1236, miR‐370 and miR‐375. RT‐qPCR was employed to verify whether circASPH and miR‐375 were dysregulated in PCOS patients. The RT‐qPCR results indicated that the expression level of circASPH in PCOS patients was obviously higher than that in control group (Figure 1A). However, the expression level of miR‐375 in PCOS patients was obviously lower than that in control group (Figure 1B). In addition, the linear regression between circASPH expression and miR‐375 expression in PCOS patients showed that the circASPH expression and miR‐375 expression was correlated negatively significantly with the p‐value 0.033 (Figure 1C). Taken together, circASPH was over‐expressed, while miR‐375 was down‐regulated in PCOS. More than that, circASPH and miR‐375 was correlated negatively.

### 3.2 Overexpression of circASPH promoted KGN cells proliferation and inhibited KGN cells apoptosis

**FIGURE 2:**
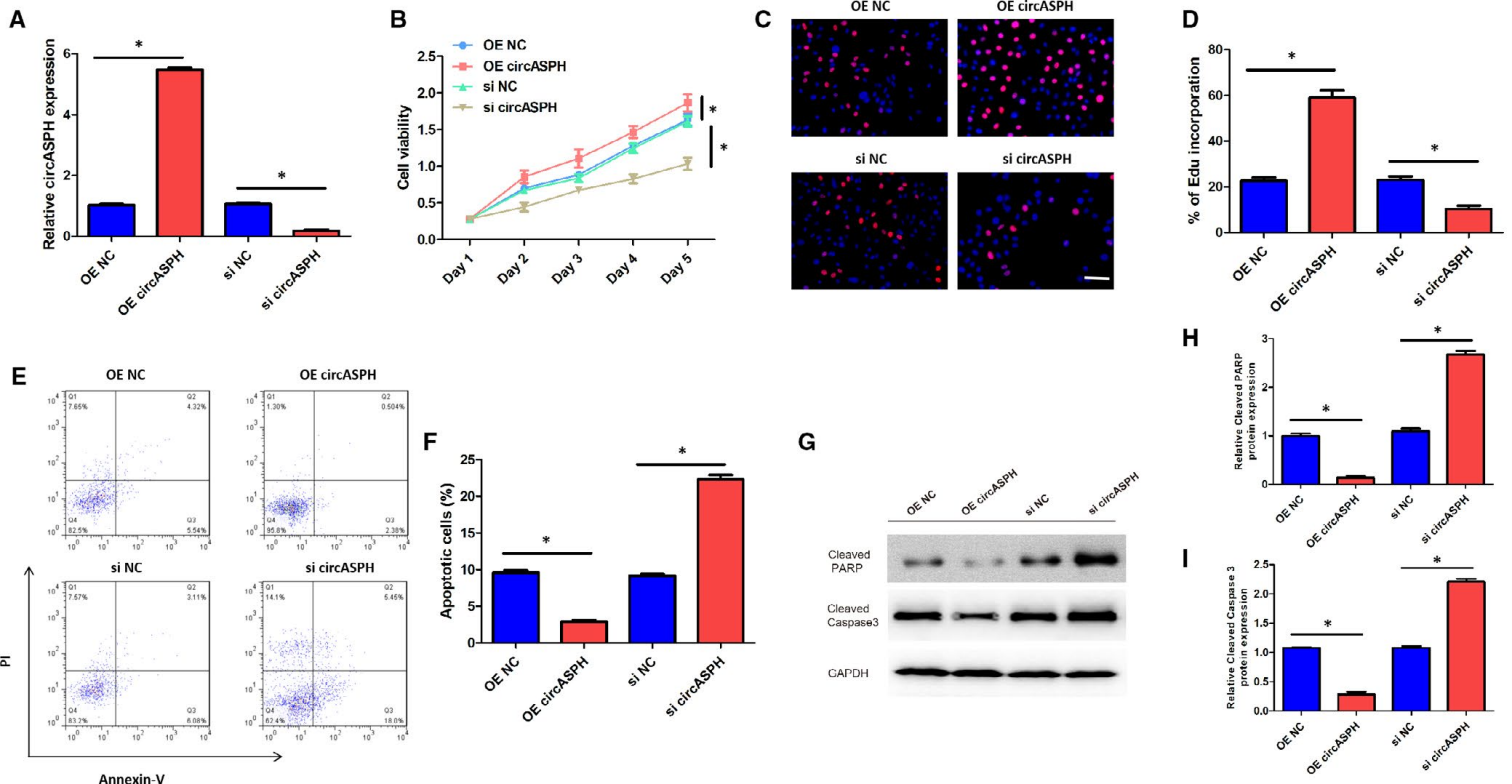
Overexpression of circASPH promoted KGN cells proliferation and inhibited KGN cells apoptosis. A, The expression levels of circASPH in KGN cells transfected with OE circASPH, OE NC, si circASPH and si NC. B, The cell viability of KGN cells transfected with OE circASPH, OE NC, si circASPH and si NC at Day 1, Day 2, Day 3, Day 4 and Day 5. C, The EdU corporation images of KGN cells transfected with OE circASPH, OE NC, si circASPH and si NC with the bar of 20 μm. D, The EdU corporation rate of KGN cells transfected with OE circASPH, OE NC, si circASPH and si NC. E, The apoptotic images of KGN cells transfected with OE circASPH, OE NC, si circASPH and si NC. F, The apoptotic rate of KGN cells transfected with OE circASPH, OE NC, si circASPH and si NC. G, H, I, The Cleaved PARP and Cleaved Caspase 3 protein expression of KGN cells transfected with OE circASPH, OE NC, si circASPH and si NC. All quantitative values were the average of three independent experiments. *P < 0.05 [Colour figure can be viewed at wileyonlinelibrary.com]

To observe the effects of circASPH on KGN cells proliferation and apoptosis, CCK‐8 assay and EdU assay were both used to double‐verify the cell viability, and apoptosis assay was used to detect the cell apoptosis rate. The expression level of circASPH in KGN and SVOG cells transfected with si circASPH‐1 or si circASPH‐2 was significantly lower than that transfected with si NC (Figure 1D and F). The cell viability of KGN and SVOG cells transfected with si circASPH‐1 or si circASPH‐2 was significantly lower than that transfected with si NC (Figure 1E and G). The expression level of circASPH in KGN cells transfected with OE circASPH was significantly higher than that transfected with OE NC and the expression level of circASPH in KGN cells transfected with si circASPH was significantly lower than that transfected with si NC (Figure 2A). CCK‐8 assay results manifested that the cell viability of KGN cells transfected with OE circASPH was significantly higher than that transfected with OE NC and the cell viability of KGN cells transfected with si circASPH was significantly lower than that transfected with si NC (Figure 2B). The EdU assay results showed that the EdU incorporation of KGN cells transfected with OE circASPH was significantly higher than that transfected with OE NC and the Edu incorporation of KGN cells transfected with si circASPH was significantly lower than that transfected with si NC (Figure 2C and D). Apoptosis assay results manifested that the cell apoptotic rate of KGN cells transfected with OE circASPH was significantly lower than that transfected with OE NC and the cell apoptotic rate of KGN cells transfected with si circASPH was significantly higher than that transfected with si NC (Figure 2E and F). Western blot analysis data revealed that the overexpression of circASPH significantly decreased the protein levels of the cleaved form of PARP and cleaved caspase 3 and silencing of circASPH significantly increased the protein levels of the cleaved form of PARP and cleaved caspase 3 (Figure 2G, H and I). Taken together, overexpression of circASPH promoted KGN cells proliferation and inhibited KGN cells apoptosis, while silencing of circASPH repressed KGN cells proliferation and enhanced KGN cells apoptosis.

### 3.3 miR‐375 was negatively regulated and directly targeted by circASPH, miR‐375 overturned the inhibition effect of circASPH silencing on KGN cells proliferation

**FIGURE 3:**
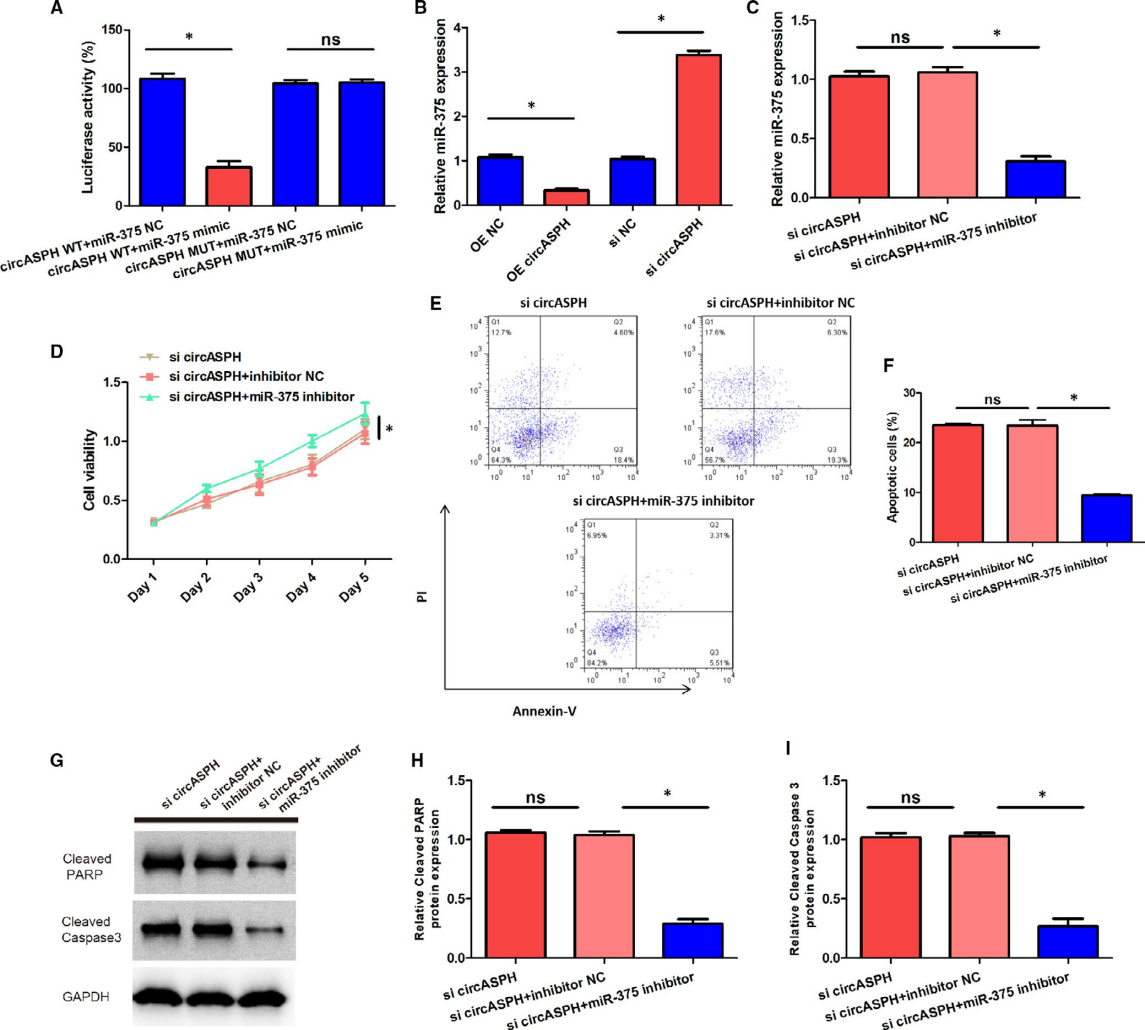
miR‐375 was negatively regulated and directly targeted by circASPH, miR‐375 reversed the inhibition effect of circASPH silencing on KGN cells proliferation. A, The luciferase activity of KGN cells transfected with circASPH WT + miR‐375 mimic, circASPH WT + miR‐375 NC, circASPH MUT + miR‐375 mimic and circASPH MUT + miR‐375 NC. B, The miR‐375 expression of KGN cells transfected with OE circASPH, OE NC, si circASPH and si NC. C, The miR‐375 expression of KGN cells transfected with si circASPH + miR‐375 inhibitor, circASPH + inhibitor NC and si circASPH. D, The cell viability of KGN cells transfected with si circASPH + miR‐375 inhibitor, circASPH + inhibitor NC and si circASPH. E, The apoptotic images of KGN cells transfected with si circASPH + miR‐375 inhibitor, circASPH + inhibitor NC and si circASPH. F, The apoptotic rate of KGN cells transfected with si circASPH + miR‐375 inhibitor, circASPH + inhibitor NC and si circASPH. G, H, I, The Cleaved PARP and Cleaved Caspase 3 protein expression of KGN cells transfected with si circASPH + miR‐375 inhibitor, circASPH + inhibitor NC and si circASPH. All quantitative values were the average of three independent experiments. *P < 0.05 [Colour figure can be viewed at wileyonlinelibrary.com]

To clarify whether miR‐375 was the downstream gene of circASPH in PCOS, we performed Dual‐luciferase reporter assay, RT‐qPCR, CCK‐8 assay and apoptosis assay. The Dual‐luciferase reporter assay results claimed that the luciferase activity of KGN cells transfected with circASPH WT + miR‐375 mimic was lower than that transfected with circASPH WT + miR‐375 NC, while the luciferase activity of KGN cells transfected with circASPH MUT + miR‐375 mimic was the same with that transfected with circASPH MUT + miR‐375 NC (Figure 3A). RT‐qPCR results indicated that the miR‐375 expression level in KGN cells transfected with OE circASPH was significantly lower than that transfected with OE NC and the miR‐375 expression level in KGN cells transfected with si circASPH was significantly higher than that transfected with si NC (Figure 3B). RT‐qPCR results also showed that the miR‐375 expression level in KGN cells transfected with si circASPH + miR‐375 inhibitor was lower both than that transfected with circASPH + inhibitor NC, which was identical with the si circASPH group (Figure 3C). CCK‐8 assay results showed that the cell viability in KGN cells transfected with si circASPH + miR‐375 inhibitor was higher than that transfected with circASPH + inhibitor NC, which was identical with the si circASPH group (Figure 3D). Apoptosis assay results manifested that the cell apoptotic rate in KGN cells transfected with si circASPH + miR‐375 inhibitor was lower than that transfected with circASPH + inhibitor NC, which was identical with the si circASPH group (Figure 3E and F). Western blot analysis data revealed that the miR‐375 inhibition significantly decreased the protein levels of the cleaved form of PARP and cleaved caspase 3 (Figure 3G, H and I). Our findings showed that miR‐375 was negatively regulated and could bind with circASPH, miR‐375 overturned the inhibition effect of circASPH silencing on KGN cells proliferation, and the promotion effect of circASPH silencing on KGN cells apoptosis. We inferred that miR‐375 was sponged by circASPH.

### 3.4 MAP2K6 was directly targeted by miR‐375 and also positively regulated by circASPH in KGN cells

**FIGURE 4:**
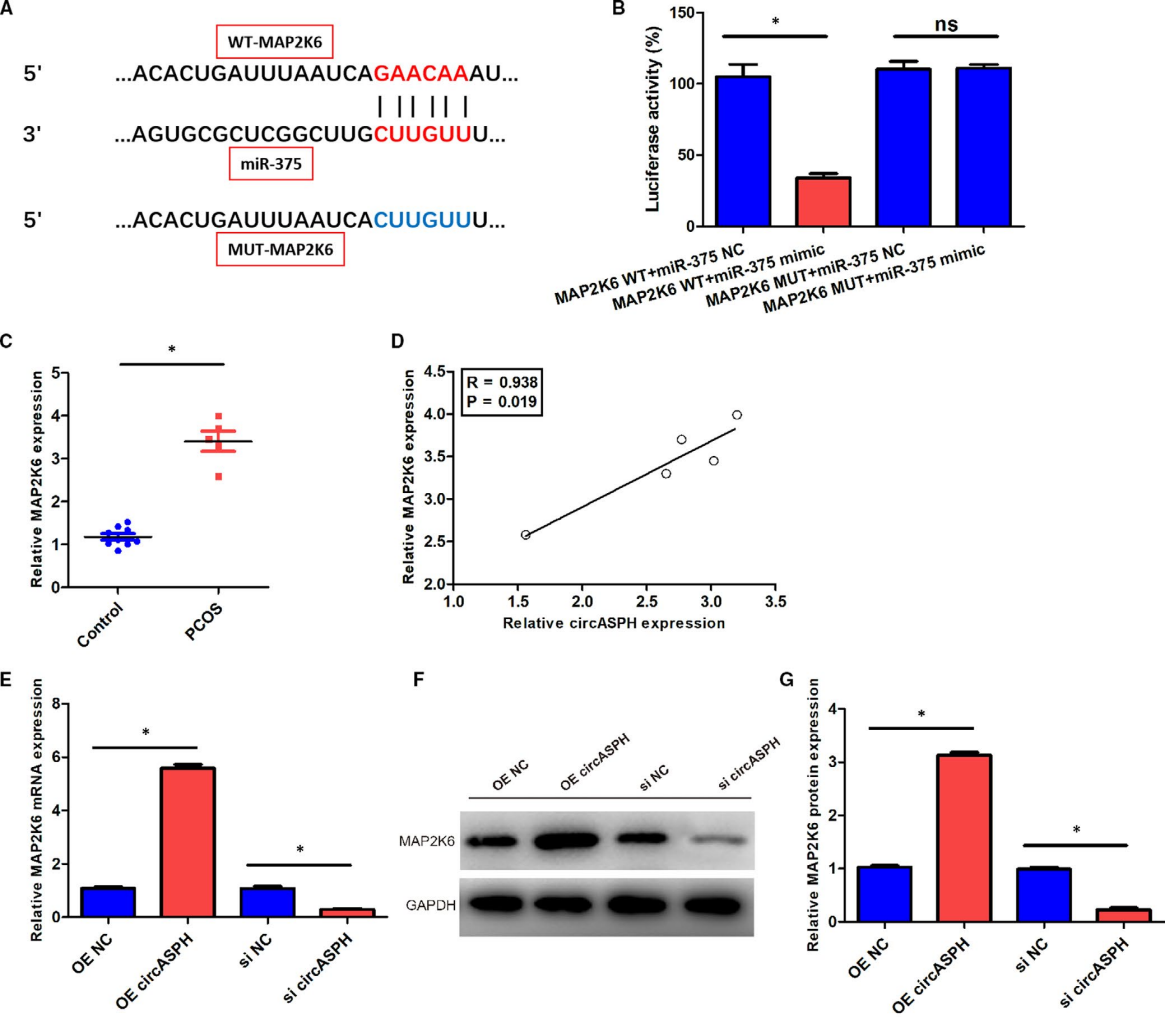
MAP2K6 was directly targeted by miR‐375, and also positively regulated by circASPH in KGN cells. A, The sequences of miR‐375, WT‐MAP2K6 and MUT‐ MAP2K6. B, The luciferase activity of KGN cells transfected with MAP2K6 WT + miR‐375 mimic, MAP2K6 WT + miR‐375 NC, MAP2K6 MUT + miR‐375 mimic and MAP2K6 MUT + miR‐375 NC. C, The expression levels of MAP2K6 in five PCOS patients and nine non‐PCOS Control group. D, The linear regression between circASPH expression and MAP2K6 expression in five PCOS patients and nine non‐PCOS Control group. E, The MAP2K6 mRNA expression of KGN cells transfected with OE circASPH, OE NC, si circASPH and si NC. F, G, The MAP2K6 protein expression of KGN cells transfected with OE circASPH, OE NC, si circASPH and si NC. All quantitative values were the average of three independent experiments. *P < 0.05 [Colour figure can be viewed at wileyonlinelibrary.com]

To explore the downstream gene of miR‐375, we employed Dual‐luciferase reporter assay, RT‐qPCR and Western blot. The sequences of miR‐375 and MAP2K6 indicated that WT‐MAP2K6 could directly bind with miR‐375, while MUT‐ MAP2K6 could not (Figure 4A). The Dual‐luciferase reporter assay results claimed that the luciferase activity of KGN cells transfected with MAP2K6 WT + miR‐375 mimic was lower than that transfected with MAP2K6 WT + miR‐375 NC, while the luciferase activity of KGN cells transfected with MAP2K6 MUT + miR‐375 mimic was the same with that transfected with MAP2K6 MUT + miR‐375 NC (Figure 4B). The RT‐qPCR results indicated that the expression level of MAP2K6 in PCOS patients was obviously higher than that in Control group (Figure 4C). Moreover, the linear regression between circASPH expression and MAP2K6 expression in PCOS patients showed that the circASPH expression and MAP2K6 expression was correlated positively significantly with the *P*‐value 0.019 (Figure 4D). RT‐qPCR and Western blot results indicated that the MAP2K6 mRNA and protein expression levels in KGN cells transfected with OE circASPH were both significantly higher than that transfected with OE NC, and the MAP2K6 mRNA and protein expression level in KGN cells transfected with si circASPH were both significantly lower than that transfected with si NC (Figure 4E, 4F and 4G). Overall, MAP2K6 was directly targeted by miR‐375 and MAP2K6 was positively regulated by circASPH in KGN cells.

### 3.5 CircASPH and MAP2K6 had a positive interaction in KGN cells

**FIGURE 5:**
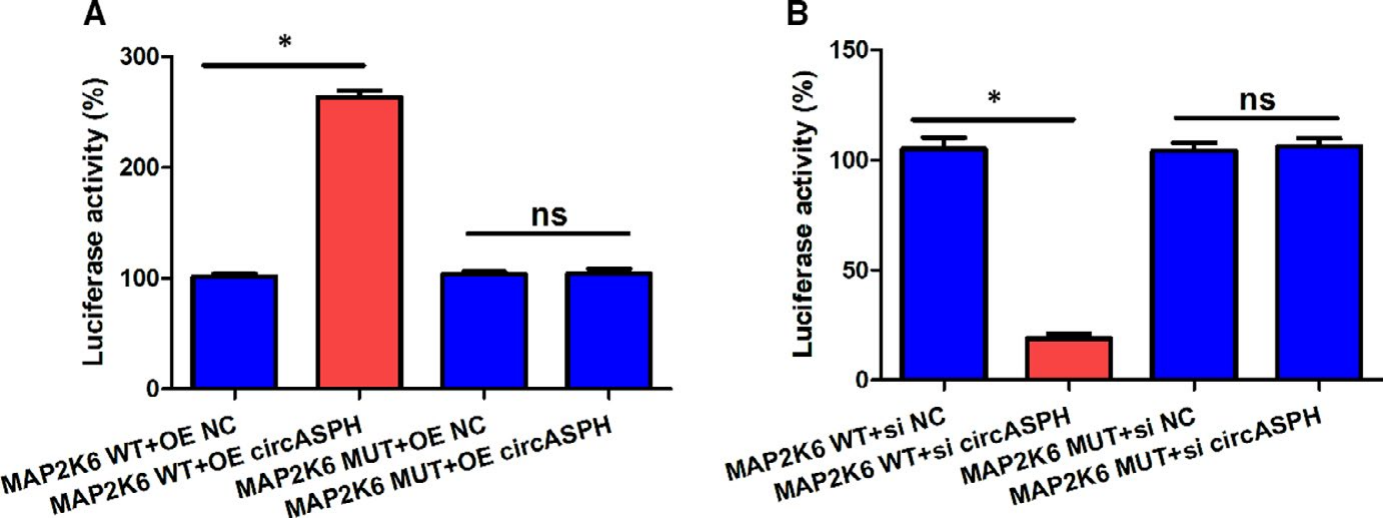
CircASPH and MAP2K6 had a positive interaction in KGN cells. A, The luciferase activity of KGN cells transfected with MAP2K6 WT + OE circASPH, MAP2K6 WT + OE NC, MAP2K6 MUT + OE circASPH and MAP2K6 MUT + OE NC. B, The luciferase activity of KGN cells transfected with MAP2K6 WT + si circASPH, MAP2K6 WT + si NC, MAP2K6 MUT + si circASPH and MAP2K6 MUT + si NC. All quantitative values were the average of three independent experiments. *P < 0.05 [Colour figure can be viewed at wileyonlinelibrary.com]

To verify the relationship between circASPH and MAP2K6 further, we performed Dual‐luciferase reporter assay in KGN cells. The Dual‐luciferase reporter assay results showed that the luciferase activity of KGN cells transfected with MAP2K6 WT + OE circASPH was higher than that transfected with MAP2K6 WT + OE NC, while the luciferase activity of KGN cells transfected with MAP2K6 MUT + OE circASPH was the same with that transfected with MAP2K6 MUT + OE NC (Figure 5A). In addition, the luciferase activity of KGN cells transfected with MAP2K6 WT + si circASPH was lower than that transfected with MAP2K6 WT + si NC, while the luciferase activity of KGN cells transfected with MAP2K6 MUT + si circASPH was the same with that transfected with MAP2K6 MUT + si NC (Figure 5B). In summary, circASPH and MAP2K6 were interacted in KGN cells, and the interaction was positive.

## 4 DISCUSSION

As an endocrine dysregulation syndrome bring varied reproductive‐related problems to women, PCOS have provoked our interest in discovering more biomarkers to serve as potential approach in PCOS diagnosis and therapy. Circular RNA have been studied to be involved in the pathogenesis of PCOS, and ceRNA network including the sponged microRNA or its target gene were also explored in PCOS Lu et al found CIRS‐126/miR‐21/PDCD4 axis was correlated with ovarian granulosa cells.^22^ Huang et al elucidated that circLDLR/miR‐1294/CYP19A1 was observed in follicle fluid.^23^

In our study, we used the human granulosa‐like tumour KGN cell line to study PCOS pathology. There were three reasons why we used KGN cells. First, it is difficult to obtain human ovarian granulosa cells in sizable amounts. Second, the primary culture system of human ovarian granulosa cells is hard to maintain. Third, KGN cells express functional follicle‐stimulating hormone receptors (FSHRs) and have steroidogenic activities similar to human ovarian granulosa cells.^24^, ^25^ We focused on the effects of circASPH in PCOS and circASPH was up‐regulated in PCOS. Silencing of circASPH repressed KGN cells proliferation and enhanced KGN cells apoptosis. Our results demonstrated that circASPH plays an oncogenic role in the pathogenesis of PCOS. In addition, miR‐375 was certificated to be targeted by circASPH, and reversed the inhibition effect of circASPH silencing on KGN cells proliferation. Moreover, MAP2K6 was found to be the downstream gene of miR‐375 and was interacted with circASPH positively. In conclusion, the circASPH/miR‐375/MAP2K6 ceRNA network in PCOS was concluded in our study.

CircASPH originated from circularization of the aspartate beta‐hydroxylase (ASPH) gene, which is located at exon 4 to exon 14, was first to be studied in lung adenocarcinoma, and it was overexpressed and regulated by HMGA2.^15^ More than that, circASPH promoted tumour cell proliferation, migration and invasion. By sponging miR‐370, circASPH modulated HMGA2 expression conversely, and served as oncogenic role through HMGA2/circASPH/miR‐370 axis.^15^ In our study, circASPH was also up‐regulated in PCOS patients, and enhanced KGN cells proliferation and inhibited KGN cells apoptosis. We found that miR‐375 was identified to be the sponging microRNA in KGN cells.

Based on the previous studies, miR‐375 have proved to be a tumour suppressor in various type of cancers essentially. MiR‐375 repressed nasopharyngeal carcinoma cells proliferation and invasion thorough targeting PDK1.^26^ In cervical cancer, miR‐375 suppressed cell proliferation, migration and invasion by interacting with AEG‐1.^27^ Overexpression of miR‐375 inhibited cell colony formation and invasion in 5‐FU‐resistant colorectal cancer cells by targeting FOXMI.^28^ Concerned to liver cancer, up‐regulated miR‐375 repressed cell growth via interacting with the downstream gene ErbB2.^29^ In gastrointestinal stromal tumour, overexpressed miR‐375‐3p attenuated cell viability and migration rate.^30^ In our study, miR‐375 was found to be sponged by circASPH and target MAP2K6, and the silencing of miR‐375 restrained PCOS development by interacting with circASPH.

Mitogen‐activated protein kinase 6 (MAP2K6) is a kind of protein kinases, belongs to MAP kinases (MAPKs) family, which contributes to cellular metabolism, cell transport, cell signalling, cell division, protein regulation and so on.^31^, ^32^ According to engaged studies, MAP2K6 was crucial to tumour cell growth, division and inflammation response.^33^ In nasopharyngeal carcinoma, MAP2K6 was associated with LIFR‐caused radioresistance.^34^ MAP2K6 was targeted by circ_016719/miR‐29c axis in neuron cell apoptosis caused by I/R.^35^ With regard to oesophageal adenocarcinoma, depleting of MAP2K6 restrained tumour cell growth.^36^ MAP2K6/p38 signalling pathway was involved in the colorectal adenocarcinoma targeted by miR‐625‐3p.^37^ In research of PCOS, MAP2K6 had already been mentioned. Nilsson et al pointed out that MAP2K6 was abnormally expressed in skeletal muscle of women with PCOS and normal group.^38^ Our study found the detailed role of MAP2K6 in PCOS. Concretely, MAP2K6 was verified to be targeted by miR‐375 and interacted with circASPH positively in KGN cells.

In general, our study revealed the oncogenic effects of circASPH in PCOS. Meanwhile, miR‐375/MAP2K6 axis was the downstream of circASPH. A ceRNA network circASPH/miR‐375/MAP2K6 was established in PCOS model, this will be an influential event for PCOS clinical diagnose and therapy.

## CONFLICT OF INTEREST

No potential conflict of interest was reported by the authors.

## AUTHOR CONTRIBUTION

**Gengxiang Wu:** Data curation (lead); Methodology (equal). **Jing Xia:** Data curation (equal); Formal analysis (equal). **Zhe Yang:** Formal analysis (equal); Methodology (equal). **Yajie Chen:** Formal analysis (equal). **Wei Jiang:** Formal analysis (equal); Methodology (supporting). **Tai‐lang Yin:** Conceptualization (equal); Supervision (equal); Writing‐review & editing (equal). **Jing Yang:** Supervision (equal); Writing‐original draft (lead).

## Data Availability

The data used to support the findings of this study are available from the corresponding author upon reasonable request.
